# Supplementary material for: Prevalence, severity and associated risk factors of anemia among human immunodeficiency virus-infected adults in Sawla General Hospital, Southern Ethiopia: A facility-based cross-sectional study
Source: PLoS One. 2023 Dec 12;18(12):e0284505. doi: 10.1371/journal.pone.0284505 (PMC10715647; doi:10.1371/journal.pone.0284505)
Supplement: Attachment — Submitted filename: Response to Reviewers.docx [file pone.0284505.s002.docx]

**Rebuttal Letter**

Dear editor and reviewers,

Greetings,

We have found your comments very important to improve our paper. Thank you very much. According to your comments, we have modified our paper. This rebuttal letter shows how we addressed your comments. For easy checkup of the document, we have put your comment with its corresponding response to editor’s comments followed by reviewer’s comments. Besides, we have uploaded the revised versions of our manuscript (one with tracked change and one without tracked change), and please kindly find them.

**Editor’s comments and their corresponding responses**

1. Conformance of our manuscript to PLOS ONE's style requirements

**Response: Fully addressed by making modifications in the document formatting.**

2. Copyright issues about location map of the study area.

**Response: Fully addressed by removing the map.**

3. Data availability issue

**Response: Fully addressed by agreeing to make the data publicly available.**

**Reviewer’s comments and their corresponding responses**

1. In the variables section the authors stated that “Anemia was the dependent variable for this study. Age, sex, education, occupation, monthly income, residence, WHO clinical stage of HIV/AIDS, CD4 cell count, VL, body mass index (BMI), drug regimen type, duration of HIV infection, and HAART, were taken as dependent variables”. But the aforementioned variables are not dependent variable rather predictors or independent variables.

**Response: Fully accepted the comment.** Based on the comment, the dependent and independent variables are now properly categorized.

In addition, I wonder if the authors of this manuscript have been measured the nutrition, disease related predictors of Anemia among PLWHIV. Since dietary intake is one of the known predictor of Anemia try to mention the common dietary practice in the study area, for instant what is the staple diet for most of the study population? Was the dietary data available? Is there any change in the dietary practice that might be differ from the other parts of Ethiopia? I advised the authors of this manuscript to clear out these things.

**Response: Fully accepted the comment.** Unfortunately, we did not assess such data. However, we accept this as an important comment for our future similar works.

2. As indicated clearly in the manuscript you conducted a pretest. If there are any amendments/modifications resulted from pretest, please mention the result obtained

**Response: Fully addressed.** Based on the pretest result, we did not made major modification to the questionnaire since we did not find major gap on it.

3. You stated that “Hosmer and Lemeshow's goodness of fitness for the logistic regression test was used to check model fitness” what was the result? Indicate the p-value

**Response: Fully addressed.** The model fitness was checked since our P value of Hosmer and Lemeshow's goodness of fitness was > 0.05

4. I also recommend to cite the references for the operational definitions

**Response: Fully addressed.** Cited.

5. The result of the multivariate analysis also needs to be corrected “(AOR: 2.32; 95% CI: 1.08-4.94, P= 0.030); being on clinical stage III or more (AOR: 4.20; 95% CI: 1.06-16.62, P= 0.041); having CD4 count below 200 cells/mm3 (AOR: 4.32; 95% CI: 2.10-8.86, P= 0.000) and BMI below 18.5 kg/m2 (AOR: 3.82; 234 95% CI: 1.83-8.00, P= 0.000). As portrayed above the p-value for CD4 count and BMI was reported as 0.000, it should not be correct you may report as <0.001 or I strongly recommend to report only (AOR with 95%CI without reporting the p-value) since the aim of this study is to identify factors associated with anemia among PLWHIV. Apart from these please display only the result of the multivariate analysis in the table. Only the eight variables which have a p-value < 0.2 logistic regression analysis and candidates of multivariate logistic regression analysis.

**Response: Fully addressed.** The sentences and numbers are corrected accordingly.

6. In the discussion section some of the reports were not completed for example Southwest Ethiopia _____ how much was the prevalence of anemia? Needs an intense revision and paraphrase to improve the readability of the manuscript…..I suggest to parphrase the following section; Various factors determine the risk of anemia among HIV-infected adults in multidimensional ways [3, 4]. In the current study, HIV-infected adults with CD4 cell count below 200 cells/mm3 (by264 4.32), BMI below 18.5 kg/m2 (by 3.82), clinical stage III or above (by 4.20), and having HIV infection for five or more years (2.32) have increased risk of anemia. Difficult to understand needs revision.

**Response: Fully addressed.** The sentences are corrected accordingly.

7. In general, this manuscript needs revision, i have some concerns about the grammar, and overall readability of the manuscript. Thus I will urge the authors of this manuscript to revise the text meticulously to fix the grammatical and typing errors and improve the overall readability of the text.

**Response: Addressed and some grammatical corrections were made.**

With kind regards,

Aklilu Alemayehu (Corresponding author)
