# Supplementary material for: Prevalence, severity and associated risk factors of anemia among human immunodeficiency virus-infected adults in Sawla General Hospital, Southern Ethiopia: A facility-based cross-sectional study
Source: PLoS One. 2023 Dec 12;18(12):e0284505. doi: 10.1371/journal.pone.0284505 (PMC10715647; doi:10.1371/journal.pone.0284505)
Supplement: Attachment — Submitted filename: Response to Reviewers.docx [file pone.0284505.s003.docx]

**Rebuttal Letter**

Dear editor and reviewer,

Greetings,

We have found your comments very important to improve our paper. Thank you very much. According to your comments, we have modified our paper. This rebuttal letter shows how we addressed your comments. For easy checkup, we have put the reviewer’s comment with its corresponding response. Besides, we have uploaded the revised versions of our manuscript (one with tracked change and one without tracked change), and please kindly find them.

**Reviewer’s comments and their corresponding responses**

1. Since it is secondary data you couldn’t find all the needed information, I understand that. But try to mention the common dietary practice of the population found in the study area. What is the staple diet of the study subjects? Is there any change in the dietary practice that might differ from the other parts of Ethiopia? Add some clarification ‘context” in the methods section to better understand the case

**Response: Fully accepted the comment.** Unfortunately, we did not assess such data. However, we accept this as an important comment for our future similar works. Furthermore, we have mentioned it under limitation part of this manuscript.

2. The result of table 3 is also not clear. You said that only variables that have an association at P < 0.2 will be recruited to the final model. “Multivariable logistic regression analysis was done for the variables with P value < 0.20 in binary logistic regression analysis to identify risk factors associated with anemia among HIV-infected adults. Based on the cutoff point, eight variables were taken as candidates for the multivariable logistic regression analysis. Finally, anemia among HIV-infected adults was significantly associated with being HIV-infected for five or more years (AOR: 2.32; 95% CI: 1.08-4.94, P= 0.030); being on clinical stage III or more (AOR: 4.20; 95% CI: 1.06-16.62, P= 0.041), having CD4 count below 200 cells/mm346 (AOR: 4.32; 95% CI: 2.10-8.86, P< 0.001) and BMI below 18.5kg/m2 47 (AOR: 3.82; 95% CI: 1.83-8.00, P< 0.001)”. But in the table, you report 14 independent variables which have no any importance i.e. variables that have a p-value > 0.2 in the univariate analysis. The purpose of variable selection in the multivariate analysis is to select clinically important and statistically significant variables while excluding unrelated variables so as to control the confounding factors. Hosmer and Lemeshow describe a purposeful selection of covariates within which an analyst makes a variable selection decision at each step of the modeling process…like Forward, Stepwise, and Backward etc. Hence, the authors of this manuscript try to fix the analysis method and report a final multivariate model which contains only 8 variables. Otherwise, clarify the case ….plausibly

**Response: Fully addressed.** Based on the reviewer’s comment, we have removed the variables that were not included for multivariable analysis. Hence, the table included only eight variables.

With kind regards,

Aklilu Alemayehu (Corresponding author)
