# Supplementary material for: Prevalence, severity and associated risk factors of anemia among human immunodeficiency virus-infected adults in Sawla General Hospital, Southern Ethiopia: A facility-based cross-sectional study
Source: PLoS One. 2023 Dec 12;18(12):e0284505. doi: 10.1371/journal.pone.0284505 (PMC10715647; doi:10.1371/journal.pone.0284505)
Supplement: Attachment — Submitted filename: comments.docx [file pone.0284505.s005.docx]

General comments

1. In abstract it is better if you donot abbreviate terms (donot use Abriavation),You shall use full form

- HIV,SPSS,SGH,ART,BMI

2. In introduction (it is to wide please reduce to 1-1*1/2 page)
3. In methodology

- Study setting
  - You shall abrievate sawla general hospital to SGH at line 97 b/c it is once abbreviated above
- Sampling technique put in sampling interval K value
- It is better if you put in figure format from total population to your sample with sampling interval

4. In data collection procedure it is better if you rewrite

- Clinical data as drug regimen, HIV stage, HAART exposure,duration of HIV
- Immunological variable as viral load and CD4

5. In operational definition pleas cite for definition HIV infected adults (15-64)

- Clearly explain frist line regimen and second line regimen you used for your research

6. In discussion it is better if you start with pathogenesis of HIV to cause anemia
7. In discussion and conclusion why you it is better to discuss for HIV infection duration, Since it have stastically significant association with anemia

Questions

1. In sample size determination why you did not use Pvalue from study done Wolayita sodo rather than Debretabor? If you use pvalue from Wolayita sodo your sample size became 356 which provide large population than your study population?

2. Why you donot included HIV infected adults who were infected after six month?

3. Why you donot used data entry software ( Epidata & epinfo) for data entry which are better for increasing quality? b/c SPSS is data analysis softaware
