# Supplementary material for: Prevalence, severity and associated risk factors of anemia among human immunodeficiency virus-infected adults in Sawla General Hospital, Southern Ethiopia: A facility-based cross-sectional study
Source: PLoS One. 2023 Dec 12;18(12):e0284505. doi: 10.1371/journal.pone.0284505 (PMC10715647; doi:10.1371/journal.pone.0284505)
Supplement: Attachment — Submitted filename: Response to Reviewers November 5 2023.docx [file pone.0284505.s006.docx]

**Rebuttal Letter**

Dear editor and reviewer,

Greetings,

We have found your comments very important to improve the quality of our paper. Thank you very much for your consideration, time, comments and email. Accordingly, we have modified our manuscript following your comments. This rebuttal letter shows how we addressed your comments in detail. For easy checkup, we have put the reviewer’s comment (numbered and italicized) with its corresponding response (shaded by color). Besides, we have uploaded the revised version of our manuscript (one with tracked change and one without tracked change), and soft copy of the original ethical clearance letter written by IRB of AMU college of Medicine and Health Science (Reference number: IRB/0203196/11). Please kindly find the uploaded documents.

**Reviewer’s comments and their corresponding responses**

1. *In abstract it is better if you donot abbreviate terms (donot use Abriavation), You shall use full form*

- HIV, SPSS, SGH, ART, BMI

**Response: Partially addressed the comment.** We have replaced SGH and SPPSS with their expanded form. But, for the interest of space we have left the commonly known abbreviations as they are (i.e. HIV, AIDS, ART and BMI), hoping the esteemed reviewer will understand the condition.

2. *In introduction (it is to wide please reduce to 1-1*1/2 page)*

**Response: Fully addressed.** Based on the reviewer’s comment, we have summarized and shortened the introduction part.

3. *In methodology*

- *Study setting*
  - *You shall abrievate sawla general hospital to SGH at line 97 b/c it is once abbreviated above*

**Response: Fully addressed.** Based on the reviewer’s comment, we have abbreviated it.

- *Sampling technique put in sampling interval K value*

**Response: Fully addressed.** Based on the calculation to determine sampling interval by considering source population and sample size, K=~ 4. Accordingly, we have described it inside the sampling technique section. Thank you!

- *It is better if you put in figure format from total population to your sample with sampling interval*

**Response: Addressed.** We have described the overall sampling technique in narration form including the sampling interval.

4. *In data collection procedure it is better if you rewrite*

- Clinical data as drug regimen, HIV stage, HAART exposure,duration of HIV
- Immunological variable as viral load and CD4

**Response: Fully addressed.** We have independently categorized these variables into clinical and immunological thematic sub-category. Thank you for this important comment.

5. *In operational definition pleas cite for definition HIV infected adults (15-64)*

**Response: Fully addressed.** We have incorporated reference for operational definition of HIV-infected adults.

- *Clearly explain* *frist line regimen and second line regimen you used for your research*

**Response: Fully addressed.** We have incorporated reference for operational definition of HIV-infected adults.

6. *In discussion it is better if you start with pathogenesis of HIV to cause anemia*

**Response: Fully addressed.** Based on the reviewer’s comment, we have started the introductory paragraph of our discussion section with pathogenesis of anemia among HIV patients.

7. *In discussion and conclusion why you it is better to discuss for HIV infection duration, Since it have stastically significant association with anemia.*

**Response: Fully addressed.** Yes, duration of HIV infection is statistically significantly associated with anemia. Hence, it is already addressed in the discussion (paragraph 6) and conclusion (paragraph 1) sections. We thank the reviewer for the reminder.

**Questions and their corresponding answers**

1. *In sample size determination why you did not use P value from study done Wolayita sodo rather than Debretabor? If you use p value from Wolayita sodo your sample size became 356 which provide large population than your study population?*

**Response: Fully addressed.** The Wolaita Sodo paper was not published during our proposal development and data collection period (before October 2019). That paper was published on PLOS ONE in October 2019. Therefore, we have used the already published paper from Debre-Tabor Hospital. We thank the reviewer for this good comment to increase sample size and closer to our study area.

2. *Why you do not included HIV infected adults who were infected after six month?*

**Response: Fully addressed.** To allow chance for finding the effect (if it exists) of ART on anemia, VL, CD4 cell count and others, we have included adults who lived with HIV for at least six months. Besides, to increase the chance of finding CD4 and VL data since these tests are often done in referral linkage that will take time for sample transportation, testing, result receiving and registration.

3. *Why you do not used data entry software (Epidata & epinfo) for data entry which are better for increasing quality? b/c SPSS is data analysis software*

**Response: Fully addressed.** Of course, we share the stand of our esteemed reviewer on the strength of epidata and epinfo to improve the quality of data during data entry. However, it is possible to directly enter into SPSS (if the data nature is less complex while taking great care to minimize error). We thank the reviewer for this critical comment and we will use these software in our future researches.

With kind regards,

Aklilu Alemayehu (Corresponding author)
